# Supplementary material for: Diffusion Coefficients and Activation Energies of Diffusion of Organic Molecules in Poly(lactic acid) Films
Source: Molecules. 2025 May 6;30(9):2064. doi: 10.3390/molecules30092064 (PMC12074079; doi:10.3390/molecules30092064)
Supplement: Supplementary file 1 [file molecules-30-02064-s001.zip › molecules-3610554-supplementary.pdf]

molecules

Diffusion Coefficients and Activation Energies of Diffusion of Organic Molecules in Poly(Lactic Acid) Films

Johann Ewender [1], Rafael Auras [2], Uruchaya Sonchaeng [2,3] and Frank Welle [1,*]

[1] Fraunhofer Institute for Process Engineering and Packaging (IVV), 85354 Freising, Germany; johann.ewender@ivv.fraunhofer.de
[2] School of Packaging, Michigan State University, East Lansing, MI *48824*, USA; aurasraf@msu.edu (R.A.); uruchaya.s@ku.ac.th (U.S.)
[3] Department of Packaging and Materials Technology, Faculty of Agro-Industry, Kasetsart University, Bangkok 10900, Thailand
* Correspondence: frank.welle@ivv.fraunhofer.de

Fourier-Transform Infrared (FTIR) Spectroscopy

The films' chemical functional groups were determined by Fourier-transform infrared (FTIR) spectroscopy (IRAffinity-1S, Shimadzu, Columbia, MD, USA) using the transmittance and attenuated total reflectance (ATR) modes. Each spectrum was recorded at a resolution of 2 1/cm and a mirror speed of 2.8 mm/s, with a total of 20 scans.

FTIR-ATR absorbance spectra of PLA-S and PLA-N (with baseline corrections) are compared in Figure S1. Despite the difference in the intensity of the peaks, the locations of the peaks appear to be the same for both films. Spectra at specific ranges of wavenumbers, as shown in Figure S2 to Figure S4, show no particular different trends besides the high or low peak intensity of one film compared to the other. However, the transmittance shown in Figure S5 shows different peak intensities between PLA-S and PLA-N, which may be attributed to differences in thicknesses.

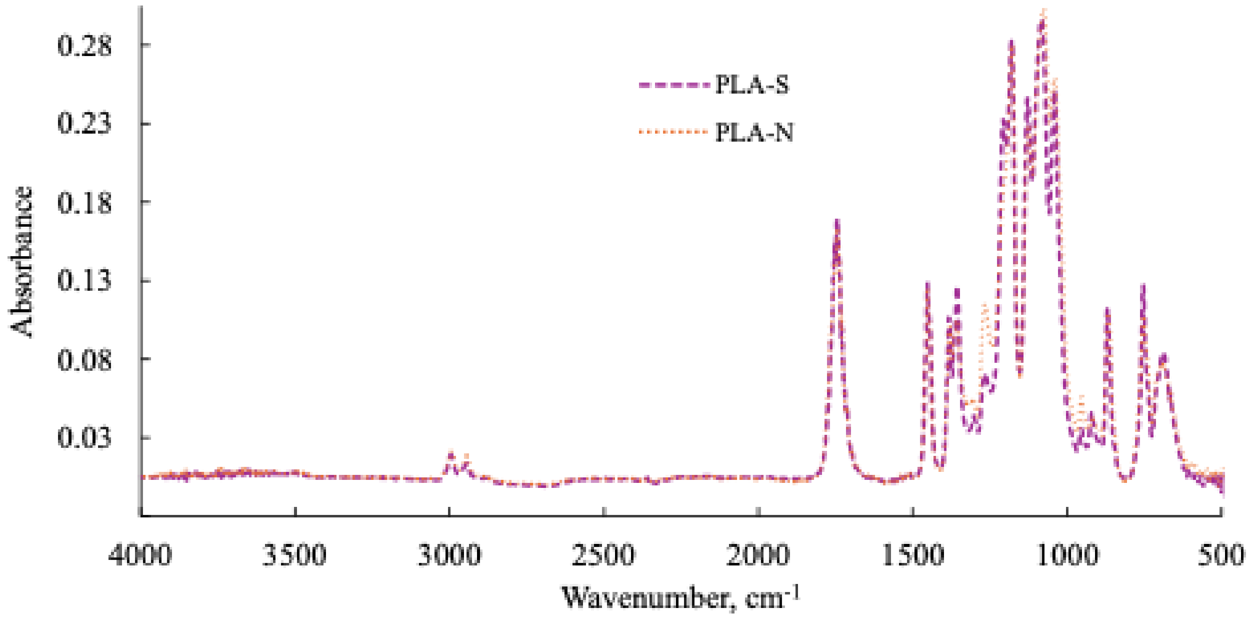

Figure S1. Full-range FTIR-ATR spectra of PLA-S and PLA-N.

 https://doi.org/10.3390/molecules30092064

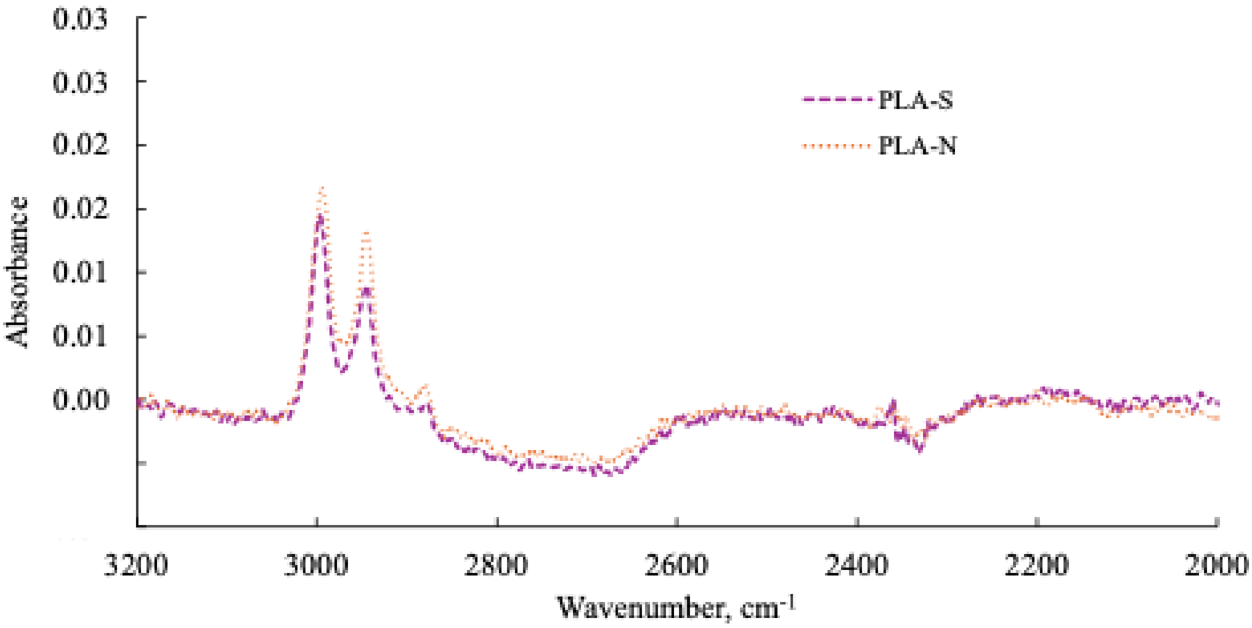

Figure S2. FTIR-ATR spectra of PLA-S and PLA-N at wavenumber 3050–2850 cm^{-1} representing -CH- stretching peaks.

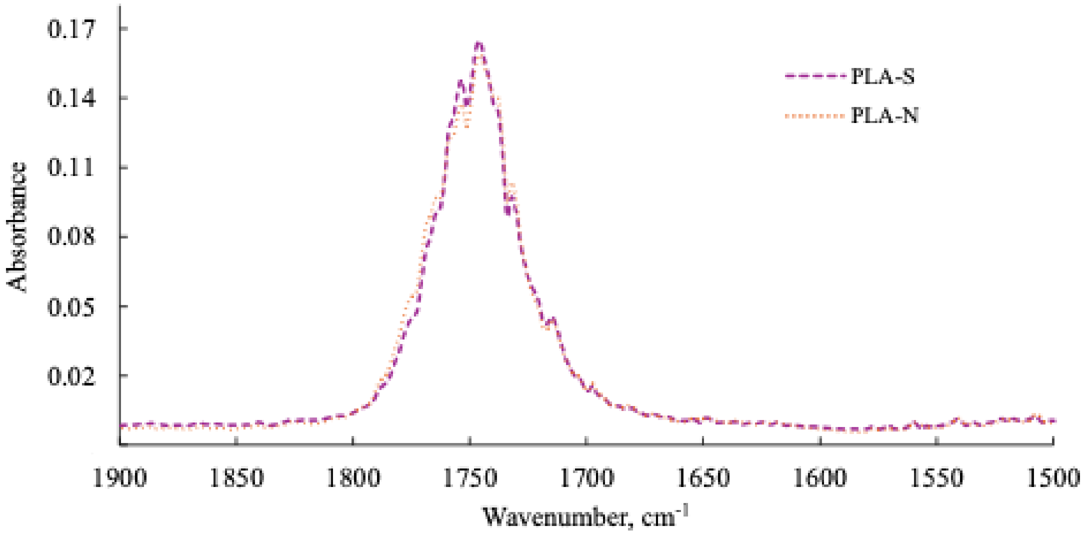

Figure S3. FTIR-ATR spectra of PLA-S and PLA-N at wavenumber 1800–1700 cm^{-1} representing -C=O- stretching peaks.

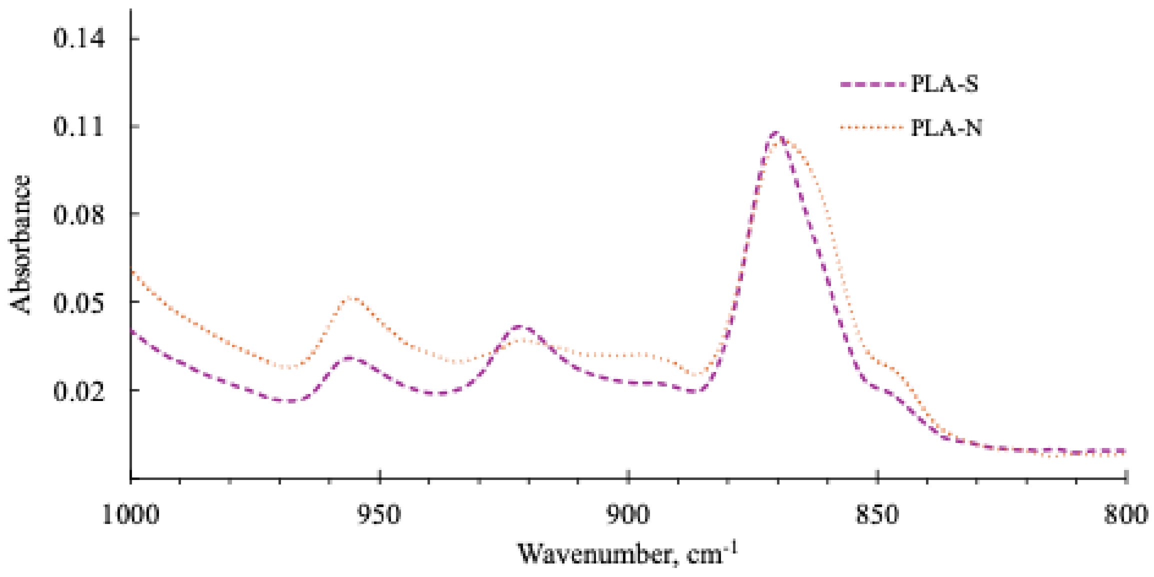

Figure S4. FTIR-ATR spectra of PLA-S and PLA-N at wavenumber 980–880 cm^{-1} representing -C-C- stretching and $-CH_3$ rocking peaks.

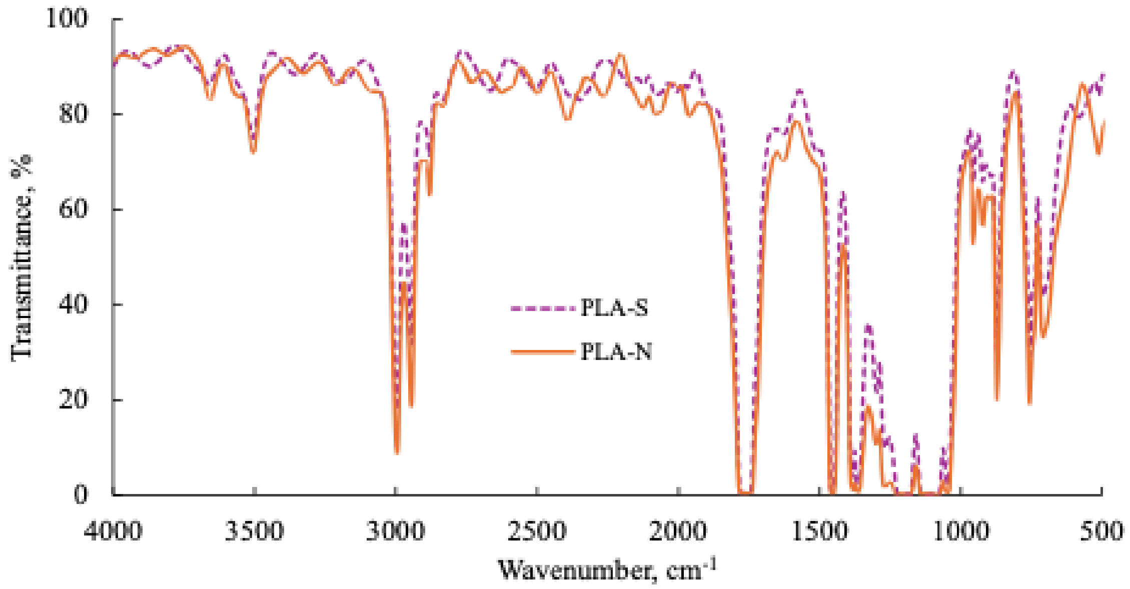

Figure S5. FTIR transmittance spectra of PLA-S and PLA-N.

Diffusion Coefficients and Activation Energies

The diffusion coefficients, D_P, and activation energies of diffusion, E_A, as well as the pre-exponential factor, D_0, determined in this study are given in Table S1 (PLA-S) and Table S2 (PLA-N). The correlations between the diffusion coefficients and the molecular volume at temperatures between 20 °C and 90 °C is given in Figure S7. The solid line is predicted from Equation 1 with parameters from Table 5. The dashed lines are predicted from ±30% in molecular volume.

Table S1. Diffusion coefficients, activation energies of diffusion and the pre-exponential factor for PLA-S

Substance	Molecular weight, g/mol	Molecular Volume, Å^3	Temperature, °C	Diffusion coefficient D_P, cm^2/s	Activation energy E_A, kJ/mol	Pre-exponential factor D_0, cm^2/s
1-propanol	60.1	70.82	70	4.64×10^{-11}		
			70	5.13×10^{-11}		
			75	1.58×10^{-10}		
n-pentane	72.2	96.16	70	3.72×10^{-12}	230.0	4.11×10^{23}
			75	1.20×10^{-11}		
			80	5.24×10^{-11}		
			85	9.65×10^{-11}		
1-butanol	74.1	87.62	70	1.13×10^{-11}	172.6	2.38×10^{15}
			70	1.28×10^{-11}		
			75	3.62×10^{-11}		
			80	7.50×10^{-11}		
			85	1.47×10^{-10}		
n-hexane	86.2	112.96	75	4.97×10^{-12}	226.8	6.88×10^{22}
			80	2.99×10^{-11}		
			85	5.92×10^{-11}		
			90	1.43×10^{-10}		
1-pentanol	88.2	104.42	70	3.45×10^{-12}	220.4	1.35×10^{22}
			70	3.66×10^{-12}		
			75	1.36×10^{-11}		
			80	4.24×10^{-11}		
			85	8.09×10^{-11}		
n-heptane	100.2	129.77	75	2.54×10^{-12}	253.9	4.32×10^{26}
			80	1.92×10^{-11}		
			85	4.26×10^{-11}		
			90	1.08×10^{-10}		
1-hexanol	102.2	121.22	75	6.55×10^{-12}		
			80	2.62×10^{-11}		
			85	6.56×10^{-11}		
n-octane	114.2	146.57	75	1.37×10^{-12}	279.3	1.56×10^{30}
			80	1.29×10^{-11}		
			85	3.24×10^{-11}		
			90	8.34×10^{-11}		
1-heptanol	116.2	138.03	75	3.65×10^{-12}		
			80	1.70×10^{-11}		
			85	5.02×10^{-11}		
n-nonane	128.3	163.37	75	8.29×10^{-13}	304.7	6.41×10^{33}
			80	9.41×10^{-12}		
			85	2.44×10^{-11}		
			90	7.48×10^{-11}		

**Substance**	**Molecular weight, g/mol**	**Molecular Volume, Å³**	**Temperature, °C**	**Diffusion coefficient D_P, cm²/s**	**Activation energy E_A, kJ/mol**	**Pre-exponential factor D_0, cm²/s**
1-octanol	130.2	154.83	75	2.30×10^{-12}		
			80	1.19×10^{-11}		
			85	3.86×10^{-11}		
n-decane	142.3	180.17	80	6.97×10^{-12}		
			85	1.90×10^{-11}		
			90	6.05×10^{-11}		
n-undecane	156.3	196.97	80	5.27×10^{-12}		
			85	1.53×10^{-11}		
			90	5.62×10^{-11}		
n-dodecane	170.3	213.78	80	4.10×10^{-12}		
			85	1.26×10^{-11}		
			90	5.15×10^{-11}		
n-tridecane	184.4	230.58	80	3.60×10^{-12}		
			85	2.06×10^{-11}		
			90	4.70×10^{-11}		
n-tetradecane	198.4	247.38	80	2.99×10^{-12}		
			85	9.20×10^{-12}		
			90	4.39×10^{-11}		

Table S2. Results for the activation energies of diffusion and the pre-exponential factor for PLA-N

**Substance**	**Molecular weight, g/mol**	**Molecular Volume, Å³**	**Temperature, °C**	**Diffusion coefficient D_P, cm²/s**	**Activation energy E_A, kJ/mol**	**Pre-exponential factor D_0, cm²/s**
methane	16.0	28.64	20	8.17×10^{-10}		
ethane	30.1	45.76	20	2.62×10^{-11}	60.6	1.47×10^{0}
			40	9.30×10^{-11}		
			50	2.09×10^{-10}		
			60	5.59×10^{-10}		
n-propane	44.1	62.56	50	9.40×10^{-12}		
			60	3.43×10^{-11}		
			70	1.52×10^{-10}		
acetone	58.1	64.74	60	3.42×10^{-11}	114.5	3.02×10^{7}
			65	6.03×10^{-11}		
			70	1.17×10^{-10}		
			75	2.13×10^{-10}		
			75	1.87×10^{-10}		
n-butane	58.1	79.36	70	3.14×10^{-11}		
methyl formate	60.1	57.16	60	2.17×10^{-10}		
			65	3.07×10^{-10}		
			70	2.92×10^{-10}		

Substance	Molecular weight, g/mol	Molecular Volume, Å^3	Temperature, °C	Diffusion coefficient D_P, cm^2/s	Activation energy E_A, kJ/mol	Pre-exponential factor D_0, cm^2/s
1-propanol	60.1	70.82	65	2.60×10^{-11}	162.0	2.81×10^{14}
			70	5.68×10^{-11}		
			75	1.60×10^{-10}		
			80	2.79×10^{-10}		
pyrrole	67.1	69.03	70	4.07×10^{-11}		
cyclopentane	70.1	85.80	70	3.28×10^{-12}	247.0	1.41×10^{26}
			75	1.36×10^{-11}		
			80	4.13×10^{-11}		
			85	1.27×10^{-10}		
tetrahydrofuran	72.1	77.98	70	1.25×10^{-11}	191.3	1.73×10^{18}
			75	3.53×10^{-11}		
			80	9.13×10^{-11}		
			85	2.06×10^{-10}		
2-butanone	72.1	81.54	60	6.01×10^{-12}	160.7	9.09×10^{13}
			65	1.31×10^{-11}		
			70	3.60×10^{-11}		
			70	2.43×10^{-11}		
			75	9.16×10^{-11}		
			75	8.10×10^{-11}		
			75	5.90×10^{-11}		
			80	2.19×10^{-10}		
			80	1.36×10^{-10}		
			85	2.77×10^{-10}		
n-pentane	72.2	96.16	70	6.74×10^{-12}	239.4	1.92×10^{25}
			75	1.93×10^{-11}		
			75	2.08×10^{-11}		
			75	2.29×10^{-11}		
			80	1.00×10^{-10}		
			80	1.09×10^{-10}		
			85	2.31×10^{-10}		
			85	1.74×10^{-10}		
			85	2.19×10^{-10}		
1,3-dioxolane	74.1	70.17	60	9.85×10^{-12}	141.4	1.47×10^{11}
			65	1.97×10^{-11}		
			70	4.20×10^{-11}		
			75	9.44×10^{-11}		
			80	1.96×10^{-10}		
			85	3.09×10^{-10}		
ethyl formate	74.1	73.97	60	2.54×10^{-11}	128.7	3.87×10^{9}
			65	4.93×10^{-11}		
			70	1.01×10^{-10}		
			75	1.85×10^{-10}		
1-butanol	74.1	87.62	65	5.14×10^{-12}	205.0	2.96×10^{20}
			70	2.14×10^{-11}		
			75	6.68×10^{-11}		
			80	1.54×10^{-10}		
			85	3.07×10^{-10}		
benzene	78.1	84.04	70	9.12×10^{-12}	212.8	2.45×10^{21}

Substance	Molecular weight, g/mol	Molecular Volume, Å^3	Temperature, °C	Diffusion coefficient D_P, cm^2/s	Activation energy E_A, kJ/mol	Pre-exponential factor D_0, cm^2/s
			75	3.13×10^{-11}		
			80	9.20×10^{-11}		
			85	2.04×10^{-10}		
pyridine	79.1	79.89	70	1.26×10^{-11}		
cyclopentanone	84.1	87.98	70	3.62×10^{-12}	242.2	2.93E+25
			75	1.43×10^{-11}		
			80	5.42×10^{-11}		
			85	1.20×10^{-10}		
cyclohexane	84.2	102.60	75	3.41×10^{-12}		
			80	1.51×10^{-11}		
			85	5.52×10^{-11}		
2-pentanone	86.1	98.34	65	2.47×10^{-12}	256.6	1.10×10^{28}
			70	1.01×10^{-11}		
			75	3.79×10^{-11}		
			75	3.24×10^{-11}		
			80	1.21×10^{-10}		
n-hexane	86.2	112.96	75	9.30×10^{-12}		
			75	1.01×10^{-11}		
			75	1.08×10^{-11}		
			80	6.27×10^{-11}		
			80	7.50×10^{-11}		
			85	1.61×10^{-10}		
			85	1.30×10^{-10}		
			85	1.41×10^{-10}		
2-methyl-1,3-dioxolane	88.1	86.75	65	3.02×10^{-12}	228.4	5.77×10^{23}
			70	9.37×10^{-12}		
			75	3.15×10^{-11}		
			80	9.33×10^{-11}		
1,4-dioxane	88.1	86.97	70	3.70×10^{-12}		
			75	1.52×10^{-11}		
			85	1.25×10^{-10}		
n-propyl formate	88.1	90.77	60	2.95×10^{-12}	191.9	3.60×10^{18}
			65	7.39×10^{-12}		
			70	2.31×10^{-11}		
			75	6.05×10^{-11}		
			80	1.39×10^{-10}		
1-pentanol	88.2	104.42	70	8.64×10^{-12}	219.3	2.43×10^{22}
			75	3.60×10^{-11}		
			80	9.45×10^{-11}		
			85	2.23×10^{-10}		
toluene	92.1	100.61	70	3.99×10^{-12}	236.6	4.81×10^{24}
			75	1.90×10^{-11}		
			85	1.38×10^{-10}		
aniline	93.1	95.33	70	3.37×10^{-12}		

Substance	Molecular weight, g/mol	Molecular Volume, Å^3	Temperature, °C	Diffusion coefficient D_P, cm^2/s	Activation energy E_A, kJ/mol	Pre-exponential factor D_0, cm^2/s
cyclohexanone	98.1	104.79	75	4.63×10^{-12}		
			80	2.20×10^{-11}		
			85	5.95×10^{-11}		
2-hexanone	100.2	115.15	70	4.51×10^{-12}	238.2	1.02×10^{25}
			75	2.26×10^{-11}		
			75	1.84×10^{-11}		
			80	7.62×10^{-11}		
			85	1.45×10^{-10}		
n-heptane	100.2	129.77	75	5.58×10^{-12}	278.9	5.12×10^{30}
			75	5.90×10^{-12}		
			75	6.23×10^{-12}		
			80	4.30×10^{-11}		
			80	5.56×10^{-11}		
			85	1.13×10^{-10}		
			85	9.28×10^{-11}		
			85	1.02×10^{-10}		
			90	3.03×10^{-10}		
n-butyl formate	102.1	107.57	65	2.02×10^{-12}	247.1	3.29×10^{26}
			70	9.02×10^{-12}		
			75	3.21×10^{-11}		
			80	8.33×10^{-11}		
1-hexanol	102.2	121.22	70	4.40×10^{-12}	241.0	2.53×10^{25}
			75	2.13×10^{-11}		
			80	6.82×10^{-11}		
			85	1.51×10^{-10}		
ethylbenzene	106.2	117.41	70	1.70×10^{-12}	246.0	6.78×10^{25}
			75	9.80×10^{-12}		
			80	4.06×10^{-11}		
			85	9.71×10^{-11}		
			90	2.01×10^{-10}		
4-vinyl cyclohexene	108.2	124.17	75	4.09×10^{-12}	246.4	4.31×10^{25}
			80	1.74×10^{-11}		
			85	6.11×10^{-11}		
			90	1.33×10^{-10}		
2-heptanone	114.2	131.95	70	2.54×10^{-12}	261.4	2.05×10^{28}
			75	1.61×10^{-11}		
			75	1.22×10^{-11}		
			80	5.71×10^{-11}		
			85	1.17×10^{-10}		
n-octane	114.2	146.57	80	3.22×10^{-11}		
			80	4.06×10^{-11}		
			85	8.59×10^{-11}		
			85	7.12×10^{-11}		
			85	7.83×10^{-11}		
			90	2.53×10^{-10}		

Substance	Molecular weight, g/mol	Molecular Volume, Å³	Temperature, °C	Diffusion coefficient D_P, cm²/s	Activation energy E_A, kJ/mol	Pre-exponential factor D_0, cm²/s
n-pentyl formate	116.2	124.37	70	4.24×10^{-12}		
			75	2.11×10^{-11}		
			80	5.52×10^{-11}		
1-heptanol	116.2	138.03	70	2.73×10^{-12}	267.5	1.66×10^{29}
			75	1.39×10^{-11}		
			80	5.13×10^{-11}		
			85	1.38×10^{-10}		
n-propylbenzene	120.2	134.21	75	5.77×10^{-12}	230.2	2.44×10^{23}
			80	2.95×10^{-11}		
			85	7.27×10^{-11}		
			90	1.63×10^{-10}		
naphthalene	128.2	128.03	75	4.31×10^{-12}	236.3	1.56×10^{24}
			80	2.51×10^{-11}		
			85	5.92×10^{-11}		
			90	1.36×10^{-10}		
2-octanone	128.2	148.75	75	1.08×10^{-11}		
			75	8.84×10^{-12}		
			80	4.35×10^{-11}		
			85	9.44×10^{-11}		
n-nonane	128.3	163.37	80	2.44×10^{-11}		
			80	3.12×10^{-11}		
			85	7.03×10^{-11}		
			85	5.08×10^{-11}		
			85	6.41×10^{-11}		
			90	2.22×10^{-10}		
n-hexyl formate	130.2	141.17	70	2.45×10^{-12}		
			75	1.36×10^{-11}		
			80	4.58×10^{-11}		
1-octanol	130.2	154.83	70	1.99×10^{-12}	279.4	7.62×10^{30}
			75	1.03×10^{-11}		
			80	4.11×10^{-11}		
			85	1.19×10^{-10}		
n-butylbenzene	134.2	151.01	75	4.35×10^{-12}	242.3	1.21×10^{25}
			80	2.32×10^{-11}		
			85	6.45×10^{-11}		
			90	1.43×10^{-10}		
methyl naphthalene	142.2	144.60	75	2.10×10^{-12}	258.0	1.35×10^{27}
			80	1.24×10^{-11}		
			85	3.77×10^{-11}		
			90	8.59×10^{-11}		
n-decane	142.3	180.17	80	1.87×10^{-11}		
			80	2.48×10^{-11}		
			85	5.84×10^{-11}		
			85	4.70×10^{-11}		
			85	5.15×10^{-11}		
			90	2.19×10^{-10}		

Substance	Molecular weight, g/mol	Molecular Volume, Å³	Temperature, °C	Diffusion coefficient D_P, cm²/s	Activation energy E_A, kJ/mol	Pre-exponential factor D_0, cm²/s
n-heptyl formate	144.2	157.97	75	9.97×10^{-12}		
			80	3.53×10^{-11}		
ethyl naphthalene	156.2	161.40	75	1.61×10^{-12}	266.2	1.75×10^{28}
			80	9.54×10^{-12}		
			85	3.10×10^{-11}		
			90	7.34×10^{-11}		
n-undecane	156.3	196.97	80	1.44×10^{-11}		
			80	2.03×10^{-11}		
			85	5.01×10^{-11}		
			85	3.88×10^{-11}		
			85	4.28×10^{-11}		
			90	1.97×10^{-10}		
n-dodecane	170.3	213.78	80	1.21×10^{-11}		
			80	1.78×10^{-11}		
			85	4.27×10^{-11}		
			85	3.30×10^{-11}		
			85	3.63×10^{-11}		
			90	1.70×10^{-10}		
n-tridecane	184.4	230.58	80	8.82×10^{-12}		
			80	1.72×10^{-11}		
			85	3.65×10^{-11}		
			85	2.86×10^{-11}		
			85	3.14×10^{-11}		
			90	1.49×10^{-10}		
n-tetradecane	198.4	247.38	80	8.54×10^{-12}		
			80	1.27×10^{-11}		
			85	3.25×10^{-11}		
			85	2.65×10^{-11}		
			85	2.82×10^{-11}		
			90	1.29×10^{-10}		
di-*iso*-propyl naphthalene	212.3	227.94	80	3.95×10^{-12}		
			85	1.41×10^{-11}		
			90	3.77×10^{-11}		

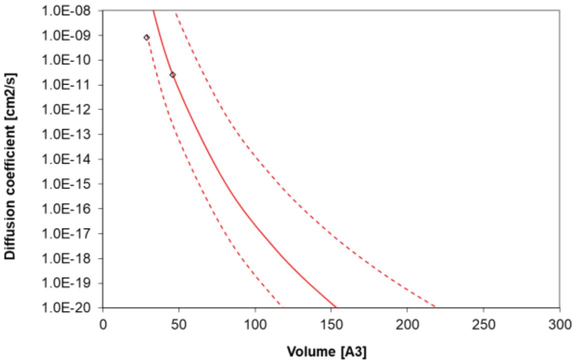

20 °C

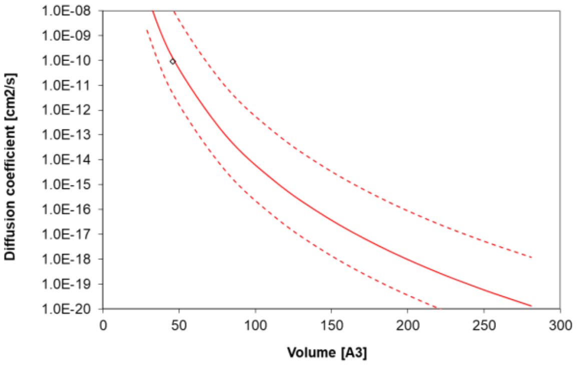

40 °C

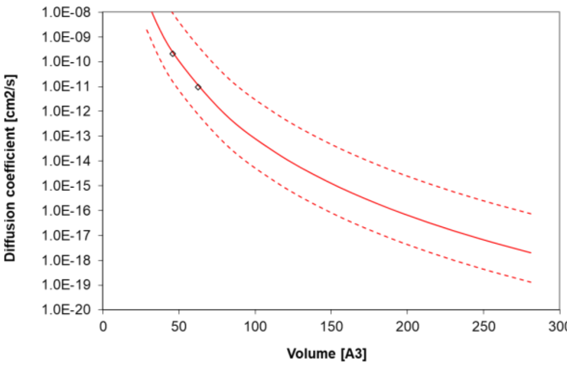

50 °C

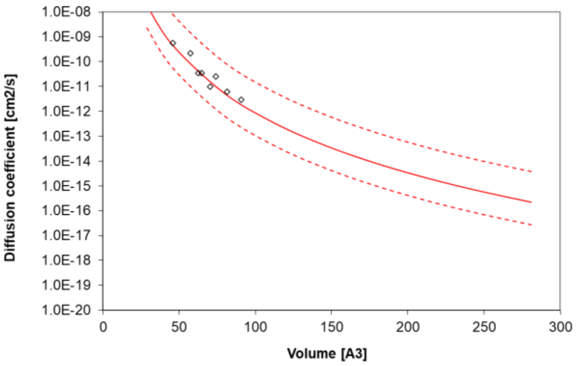

60 °C

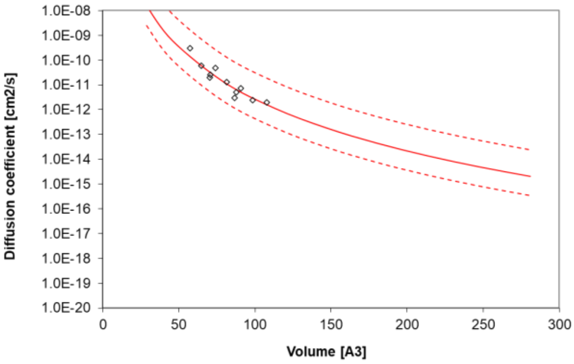

65 °C

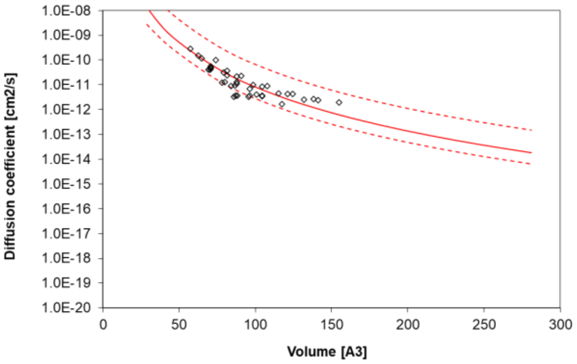

70 °C

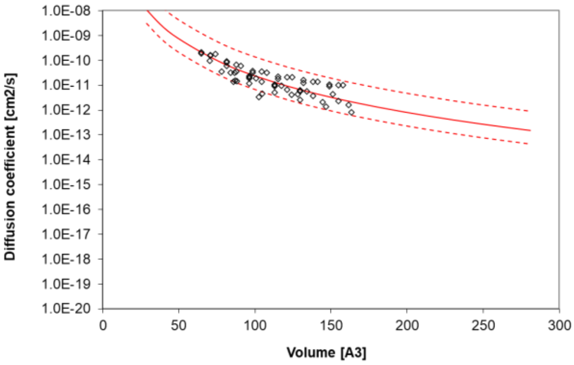

75 °C

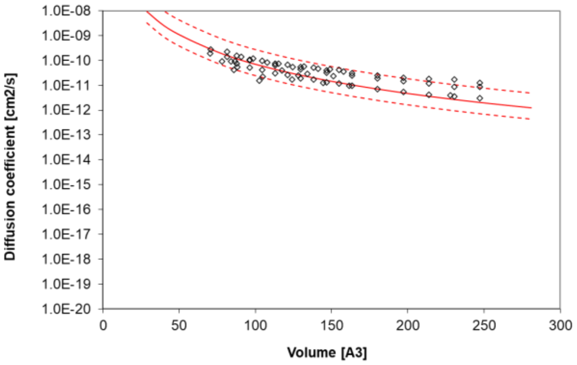

80 °C

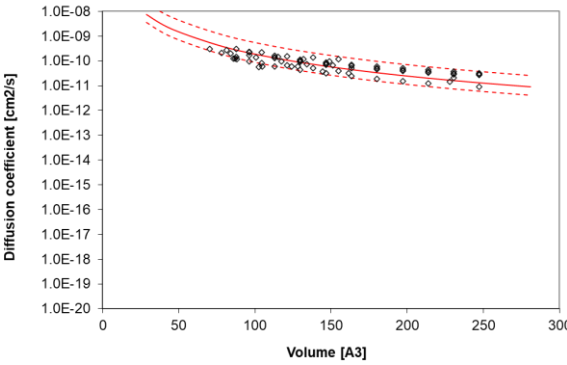

85 °C

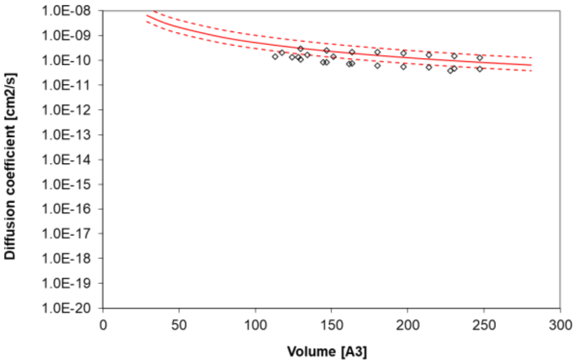

90 °C

Figure S6. Correlation between the diffusion coefficients and the molecular volume at temperatures between 20 °C and 90 °C. Solid line: predicted from Equation 1 with parameters from Table 5, dashed lines ±30% in volume

Disclaimer/Publisher's Note: The statements, opinions and data contained in all publications are solely those of the individual author(s) and contributor(s) and not of MDPI and/or the editor(s). MDPI and/or the editor(s) disclaim responsibility for any injury to people or property resulting from any ideas, methods, instructions or products referred to in the content.
